# Catalytic Production of Oxygenated and Hydrocarbon Chemicals From Cellulose Hydrogenolysis in Aqueous Phase

**DOI:** 10.3389/fchem.2020.00333

**Published:** 2020-05-05

**Authors:** Haosheng Xin, Xiaohong Hu, Chiliu Cai, Haiyong Wang, Changhui Zhu, Song Li, Zhongxun Xiu, Xinghua Zhang, Qiying Liu, Longlong Ma

**Affiliations:** ^1^Guangzhou Institute of Energy Conversion, Chinese Academy of Sciences, Guangzhou, China; ^2^CAS Key Laboratory of Renewable Energy, Guangzhou, China; ^3^Guangdong Provincial Key Laboratory of New and Renewable Energy Research and Development, Guangzhou, China; ^4^University of Chinese Academy of Sciences, Beijing, China; ^5^Nano Science and Technology Institute, University of Science and Technology of China, Suzhou, China; ^6^Dalian National Laboratory for Clean Energy, Dalian, China

**Keywords:** cellulose, hydrolysis, hydrogenolysis, biorefinery, catalysis, aqueous phase

## Abstract

As the most abundant polysaccharide in lignocellulosic biomass, a clean and renewable carbon resource, cellulose shows huge capacity and roused much attention on the methodologies of its conversion to downstream products, mainly including platform chemicals and fuel additives. Without appropriate treatments in the processes of cellulose decompose, there are some by-products that may not be chemically valuable or even truly harmful. Therefore, higher selectivity and more economical and greener processes would be favored and serve as criteria in a correlational study. Aqueous phase, an economically accessible and immensely potential reaction system, has been widely studied in the preparation of downstream products of cellulose. Accordingly, this mini-review aims at making a related summary about several conversion pathways of cellulose to target products in aqueous phase. Mainly, there are four categories about the conversion of cellulose to downstream products in the following: (i) cellulose hydrolysis hydrogenation to saccharides and sugar alcohols, like glucose, sorbitol, mannose, etc.; (ii) selective hydrogenolysis leads to the cleavage of the corresponding glucose C-C and C-O bond, like ethylene glycol (EG), 1,2-propylene glycol (PG), etc.; (iii) dehydration of fructose and further oxidation, like 5-hydroxymethylfurfural (HMF), 2,5-furandicarboxylic acid (FDCA), etc.; and (iv) production of liquid alkanes *via* hydrogenolysis and hydrodeoxygenation, like pentane, hexane, etc. The representative products were enumerated, and the mechanism and pathway of mentioned reaction are also summarized in a brief description. Ultimately, the remaining challenges and possible further research objects are proposed in perspective to provide researchers with a lucid research direction.

## Introduction

To alleviate the adverse impact caused by excessive consumption of traditional fossil fuels, finding a green-renewable-sustainable resource on Earth that could be regarded as a replacement of fossil has aroused much attention nowadays. Meanwhile, this resource that we are striving to find could not only meet the global growing energy needs but also demonstrate the potential of promising block for application in the fields of crucial medicines, high value-added chemicals, and functional materials, etc. (Delbecq et al., 2018). Lignocellulosic biomass, the most promising renewable resource on the planet, has already been integrated into the blueprint of energy system for its high economic and practical application value (Den et al., 2018). In common, lignocellulosic biomass consists of 10–25% lignin, 20–35% hemicellulose, and 35–50% cellulose. Serving as the most abundant component of lignocellulosic biomass, cellulose plays a vital role not only in the field of utilizing of renewable resource but also in providing more possibilities for the production of a battery of high value-added chemicals (Wang et al., 2019). Therefore, how to efficiently catalyze the conversion of cellulose to desirable products by hydrolysis and subsequent reactions is challengeable and has become the meritorious object pursued by researchers.

Well-known that the cellulose molecules in biomass are formed by the glucose units linked by β-1,4 glycosidic bonds and the molecular chains are connected to each other through numerous hydrogen bonds (Singh et al., 2016). Therefore, the primary premise of being able to take full advantage of cellulose is to break those β-1,4 glycosidic bonds and hydrogen bonds. At the outset, direct pyrolysis of cellulose has been paramount considered as an efficient means to obtain target fuels and chemicals (Atalla and Vanderhart, 1984). The degree of polymerization of cellulose begins to decrease with the increase of pyrolysis temperature; meanwhile, hydrogen bonds (Xin et al., 2015) and β-1,4 glycosidic bonds (Yu et al., 2012) are successively broken. In view of the effect of β-1,4 glycosidic linkage, multiformity of end-products and complexity of reactions in the research of cellulose pyrolysis, cellulose model compounds (including cellotriose, cellobiose, glucose, etc.) are usually adopted as selection objects (Paine et al., 2007; Mayes et al., 2014; Zhang et al., 2015a; Zhang M. et al., 2015; Gao Z. et al., 2019). Moreover, the degree of polymerization and positions and orientation of glycosidic linkages about organic product speciation were also investigated among different model compounds when comparing to the elementary products distribution after pyrolysis of glucose and its oligomers (Patwardhan et al., 2009). Researchers found that the positions and orientation of glycosidic linkages caused an insignificant effect on elementary products distribution except for the case of dextran, the elementary product—levoglucosane yield trend follows the order: monosaccharide < disaccharides < oligosaccharides < polysaccharides. Although pyrolysis is a capable method in the field of directly converting cellulose, it is limited to its single conversion means and more complicated uncontrollable reactions, making it difficult to obtain a specific target product directly through a specified reaction path. Therefore, researchers are also promoted to explore other effective ways to convert cellulose to the target products.

In the past few decades, researchers have endeavored to overcome the obstacles in the process of cellulose biorefinery, such as intrinsic robust, complex, and heterogeneous structure of cellulose, so as to depolymerize cellulose into intermediary monosaccharide and further transformation to other high value-added chemicals. Pursuing the philosophy of efficient conversion of cellulose and hydrolyzed monosaccharide, organic solvent, biphasic solvent, ionic liquid, and aqueous-phase solvent systems were adopted and confirmed that high selectivity and yield of downstream products could be obtained (Ji et al., 2008; Van Putten et al., 2013; Wang et al., 2015; Mariscal et al., 2016; Zhang Z. et al., 2017). For instance, a biphasic system made up of water and biomass-derived γ-valerolactone (GVL) has achieved a great progress in the hydrolysis of lignocellulosic biomass into various carbohydrates by disrupting the hydrogen-bond network or decreasing the transparent activation energy (Mellmer et al., 2014a; Li W. et al., 2017; Xin et al., 2017; Zhang T. et al., 2017; Zhang et al., 2019). It is also demonstrated that 5-hydroxymethylfurfural (HMF) is inclined to be steady due to the existence of GVL–water biphasic solvent system and reduce the occurrence of side reactions, thereby promoting the reaction toward the target product. In addition, not confined to monosaccharide, cellulose can also be directly converted into HMF with higher yield in tetrahydrofuran (THF)/H_2_O-NaCl biphasic system using Sn-Mont catalyst and THF–seawater biphasic system even without any acid added (Wang et al., 2012; Li et al., 2018). In essence, the yield could be enhanced because HMF can be simultaneously extracted into organic phase after formation in a biphasic system, which could maintain HMF in a steady state and avoid the occurrence of some side reactions. And further studies also confirmed that the reaction kinetics can be influenced by aprotic organic solvents (e.g., GVL, THF) in the way of transforming the stabilization of the acidic proton vs. the protonated transition state, resulting in enormous increases of reaction rates and product selectivity in acid-catalyzed systems (Mellmer et al., 2014b, 2018). Moreover, ionic liquids as novel catalysts have shown its giant potential in the conversion of various raw biomass materials and their derivatives that could accord with the demand of green chemistry (Zhang et al., 2005; Li and Zhao, 2007; Li et al., 2008; Zavrel et al., 2009). For example, an ionic liquid for cellulose hydrolysis named 1-butyl-3-methylimidazolium chloride [(C_4_mim)Cl] has been verified to accelerate reaction rates so that facilitating the hydrolysis of cellulose dramatically under atmospheric pressure. In [C_4_mim]Cl solvent, cellulose could completely dissolve and then form a homogeneous solution, which made the H^+^ more accessible to the β-1,4 glycosidic bonds so as to promote the reaction process (Li and Zhao, 2007). In addition, two Brønsted acidic ionic liquid catalysts [1-(3-proylsulfonic)-3-methylimidazolium chloride and 1-(4-butylsulfonic)-3-methylimidazolium chloride] were successfully applied to the further conversion to furanic biocrude products including HMF after cellulose hydrolysis, and several products formed from aldol condensation of HMF with one to three acetone molecules and some other HMF ether products (Amarasekara and Reyes, 2019).

Even a series of organic solvents and ionic liquids reported previously has exhibited a certain level of activity in the conversion of biomass raw materials and derived model compounds, aqueous-phase solvent has the most practical application value especially in economics to obtain downstream products in view of the difficulty of separation recovering the organic solvents after reaction and expensive price of ionic liquids.

## Categories of Cellulose Conversion

Cellulose conversion includes both conversion between different cellulose forms (such as microcrystalline cellulose, cellulose nanocrystals, cellulose acetate, cellulose nitrate could be obtained) and deep cellulose degradation to small-molecule chemicals. This review would focus on the deep cellulose degradation to representative small molecular chemicals in aqueous phase, which is also different from those reviews about introducing cellulose conversion in the field of catalyst, reaction pathway, or organic solvent effects (Shuai and Luterbacher, 2016; Jing et al., 2019; Pang et al., 2019). Cellulose can be catalytically converted into various chemicals as the established reaction paths under aqueous-phase reaction atmosphere, sugars (glucose, fructose, sorbitol, mannitol, etc.), furan compounds [HMF, N,N-dimethylformamide (DMF), 2,5-furandicarboxylic acid (FDCA), etc.], alcohols [ethylene glycol (EG), ethanol, erythritol, 1,2-propanediol, etc.], liquid hydrocarbons (pentane, hexane, methylcyclopentanone, etc.), phenols and other pharma [hydroxyacetone (HA), 1-hydroxy-2-butanone (HB), etc.], or polymer building blocks are available.

**Scheme 1:**
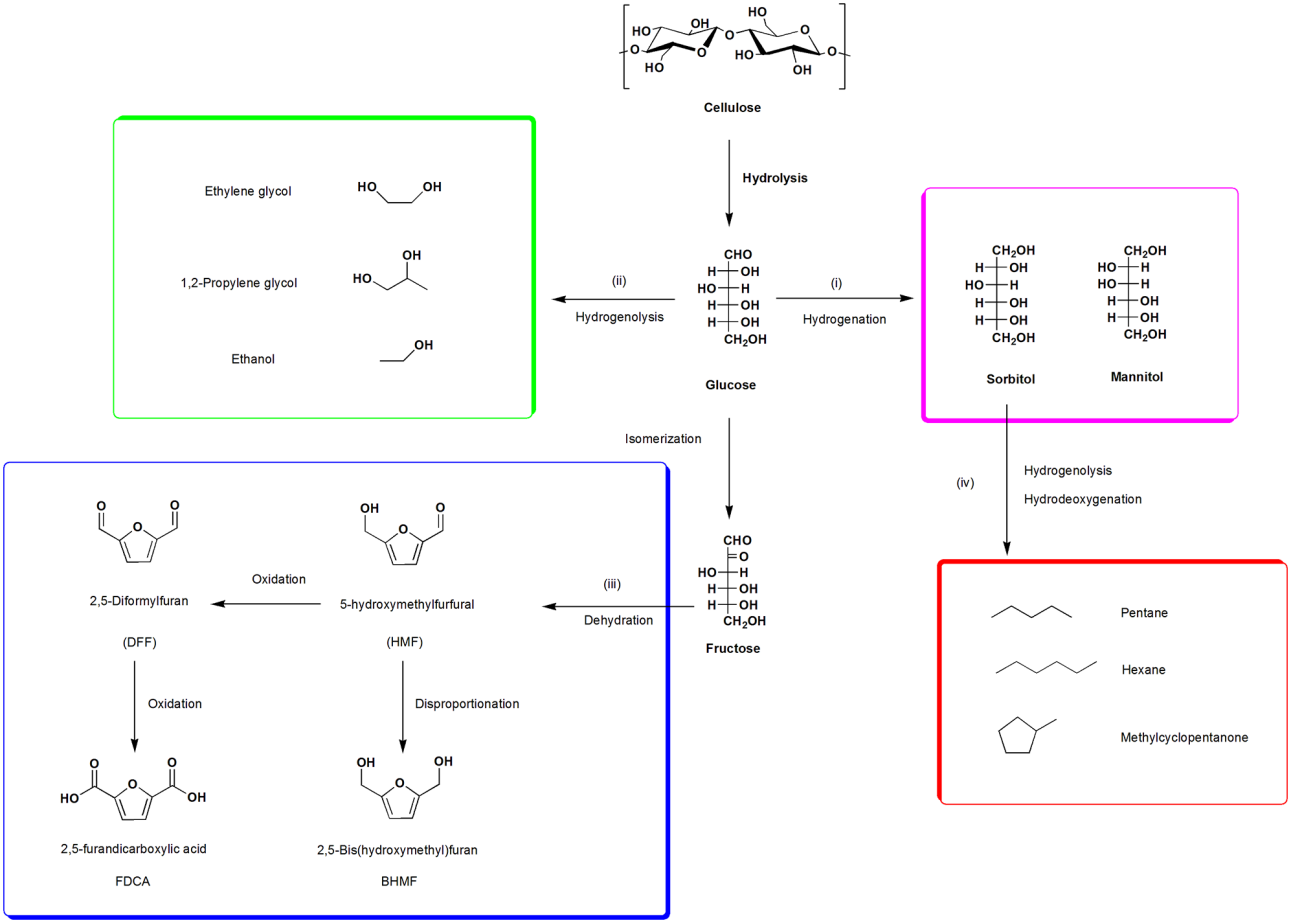
Conversion of cellulose to platform chemicals and fuels.

In summary, there are mainly four categories of reactions during biorefinery and catalytic process of cellulose degradation in aqueous phase are summed up hereinafter (Scheme 1): (i) cellulose hydrolysis and hydrogenation to sugar alcohols; (ii) selective hydrogenolysis of glucose to alcohols by fracturing the corresponding C-C and C-O bond; (iii) further dehydration and hydrogenation of fructose (glucose isomerization) to HMF and its derivatives; (iv) *via* hydrogenolysis and hydrodeoxygenation to liquid alkanes. Some other reaction types may be also referred, including but not limited to isomerization, epimerization, oxidation, aldol condensation, or hydration, and all the reaction types abovementioned are carried out in aqueous phase. In addition to those abovementioned, a very hot topic about using enzymes as green and sustainable catalysts for the processing of cellulose conversion and the production of derived green solvents would also be introduced, such as bioethanol, levoglucosenone, and dihydrolevoglucosenone (cyrene).

### Cellulose Hydrolysis Hydrogenation to Saccharides and Sugar Alcohols

#### Polysaccharides

For a better understanding of the cellulose hydrolysis process, high degree of polymerization of cellulose is hydrolyzed to cello-oligosaccharides firstly, which are biologically important molecules that can be directly used in agricultural and food industries that are made up of 10 or less glucose units linked by β-1,4 glycosidic bonds and could be degraded to a single glucose unit fleetly. Homogeneous catalysts could dissolve and hydrolyze cellulose. Eighty-five percent H_3_PO_4_ solution and 3.8% HF/SbF_5_ were tested for the synthesis of cello-oligosaccharides from cellulose (Liebert et al., 2008; Martin-Mingot et al., 2012). Although acceptable activities were achieved, the difficulty for catalysts recycling, water pollution problems, and certain operational risk limited the application of such catalysts. Subsequently, carbon materials containing oxygenated functional groups as heterogeneous catalysts are stabilized under the hydrothermal and high-pressure conditions requested for cellulose hydrolysis (Kobayashi et al., 2013; Charmot et al., 2014; Zhao et al., 2014). Reaction mechanisms reveal that cellulose adsorbed on the carbon material surface primarily relies on CH-π and hydrophobic interactions, and then the acidic functional group designed on the catalyst surface acting on the β-1,4 glycosidic bonds resulting in the hydrolysis of cellulose to cello-oligosaccharides (Yabushita et al., 2014; Kobayashi et al., 2015). Food-grade activated carbon with weak acidity after being oxidized in air shows a good ability in the field of cellulose conversion to cello-oligosaccharides, 56% yield of target product were obtained in batch reactor with 40 ml distilled water under 453 K after 20 min (Chen et al., 2019). Consistent with the literatures abovementioned, with good adsorption of catalyst to cellulose, acidic functional groups on activated carbon surface and high space velocity are essential to make a contribution to maximize the product yield.

#### Glucose

Besides polysaccharides, glucose is the target product during the conversion of cellulose as it is a significant precursor to all kinds of valuable chemicals and fuels (Alonso et al., 2010). Hence, the efficient hydrolysis of cellulose to glucose is one of the most important subjects with low environmental impact in green and sustainable chemistry. Homogeneous sulfuric acid catalyst (Rinaldi and Schüth, 2009), cellulase enzymes (Kim and Hong, 2001), sub- or supercritical water (Adschiri et al., 1993), and heterogeneous solid catalysts (Rinaldi and Schüth, 2009) were successfully applied to the hydrolysis of cellulose, but homogeneous acid catalyst is highly corrosive and neutralizing treatment is required after reaction in order to avoid environmental problems; cellulose enzymes show a low reaction rate with high costs, and it is difficult to recover the enzyme from the mixtures after reaction; reaction apparatuses with high quality are desired due to the harsh conditions under sub- or supercritical water, and the thermal instability of glucose at high temperature caused the low selectivity of this method. Thereby, heterogeneous solid catalysts are gaining increasing attention and would be favorable for cellulose hydrolysis because solid catalysts not only can be applied under a wide range of reaction conditions and pH tolerance but also can be easily separated from the mixture after reaction with good cycle performance (Jun et al., 2000; Kitano et al., 2009; Yamaguchi et al., 2009). In the selective hydrolysis of cellulose to glucose, a sulfonated activated-carbon catalyst (AC-SO_3_H) was synthesized and showed an excellent catalytic activity and high selectivity (over 90%) of glucose compared with H-morderite, H-ZSM5, AC, sulfated zircoria, Amberlyst 15, and other solid materials, which turns out that the high hydrothermal stability and the prominent catalytic property are attributed to the surface acid sites of SO_3_H functional groups and the intrinsic hydrophobic planes (Onda et al., 2008). Sulfonic groups might become unstable and leach into the water under high temperature and pressure that are required in case of further conversion to target chemicals and fuels, which may lead to a decrease in catalyst activity and the yield of the target products. In the meantime, solid materials supported various metal (Pt, Ru, Ni, etc.) catalysts were also investigated with respect to the degradation of cellulose (Fukuoka and Dhepe, 2006; Luo et al., 2007; Deng et al., 2009). A water-tolerant solid catalyst Ru/CMK-3 was successfully synthesized by combining mesoporous carbon material (CMK-3) and metal Ru, which provided high glucose yields and turnover numbers (TONs) for the hydrolysis of cellulose in pure aqueous phase (Kobayashi et al., 2010). The results of X-ray diffraction (XRD) and transmission electron microscopy (TEM) indicate that Ru is uniformly dispersed on the surface of CMK-3 and not reduced to form zero-valent nanoparticles thoroughly, without cramming the CMK-3 pores. There is a synergistic effect between CMK-3 and Ru, in which CMK-3 plays a vital role for the hydrolysis of cellulose to intermediate oligosaccharides while Ru promotes the further conversion of oligosaccharides into glucose.

#### Fructose

Generally, fructose is derived from the isomerization of glucose obtained after the hydrolysis of cellulose in the presence of enzymes, homogeneous acids, bases, or Lewis acidic sites, which plays a key role in the way of producing platform chemicals and fuels (Lee and Hong, 2000; Tanase et al., 2001; Moliner et al., 2010; Liu C. et al., 2014). Recently, the catalytic performance of macroporous niobium phosphate (NbP) supported by MgO catalysts for isomerization of glucose to fructose was investigated and shows high efficiency in water and air atmosphere (Gao D. et al., 2019). The TON (defined as the number of moles of fructose formed per mole of basic sites on the fresh catalyst when the yield of fructose reached maximum value) values increased with the increase of MgO content from 20 to 60 wt%. And the surface characteristics of MgO were obviously improved while loading on the synthesized porous niobium phosphate, the distribution and amounts of basic sites and the resistance to water are comprised. However, noting that the yield of fructose was obviously decreased because of the mediocre cycle performance of MgO/NbP after first reaction without regeneration, thus the practical application value is limited. A heterogeneous bifunctional solid catalyst (UiO-66-MSBDC, substituted the organic linker of the zirconium organic framework UiO-66 with 2-monosulfo-benzene-1,4-dicarboxylate partially) with Brønsted and Lewis acid sites was successfully prepared and shows good stability and selectivity for glucose isomerization to fructose in deionized water (Oozeerally et al., 2018). The Lewis acidity can be affected while modification. UiO-66 was modified by MSBDC, attributed to the formation of more defective materials, and the nearby electron-withdrawing groups are enhanced in the presence of Zr^4+^, consistent with previous literature reports (Degirmenci et al., 2011). Some representative works abovementioned on the preparation of saccharides and sugar alcohols are summarized in Table 1.

**Table 1:** Representative work on the preparation of saccharides and sugar alcohols.

**Entry 1**	**Substrate**	**Solvent**	**Catalyst**	**Temperature (^**°**^C)**	**Time**	**Yield (%)**	**References**
1	Cellulose	H_2_O	AC-Air	180	20 min	Cello-oligosaccharides:54	Chen et al., 2019
2	Cellulose	H_2_O	AC-SO_3_H	150	24 h	Glucose:40.5	Onda et al., 2008
3	Cellulose	H_2_O	Amberlyst-15	150	24 h	Glucose:26.7	Onda et al., 2008
4	Cellulose	H_2_O	Pt/γ-Al_2_O_3_	190	24 h	Sorbitol:25 Mannitol:6	Fukuoka and Dhepe, 2006
5	Cellulose	H_2_O	SiO_2_-SO_3_H	150	10 h	Glucose:56.6	Zhu et al., 2014
6	Cellulose	H_2_O	Ru/SiO_2_+SiO_2_-SO_3_H	150	10 h	Sorbitol:43.3 Mannitol:14.7	Zhu et al., 2014
7	Cellulose	H_2_O	Ru/SiO_2_+SO_3_H	150	10 h	Sorbitol:61.2 Mannitol:6.9	Zhu et al., 2014
8	Glucose	H_2_O	Ru/SiO_2_+SO_3_H	150	30 min	Sorbitol:97.5	Zhu et al., 2014
9	Glucose	H_2_O	Ru:Ni/MCM-48	120	90 min	Sorbitol:31	Romero et al., 2017
10	Glucose	H_2_O	MgO/NbP-500	120	30 min	Fructose:24.6	Gao D. et al., 2019
11	Glucose	H_2_O	MgO/ Al_2_O_3_	120	30 min	Fructose:27.1	Gao D. et al., 2019

#### Sorbitol and Mannitol

When some metal catalysts with hydrogenation effect is added to the reaction, during the hydrolytic process of cellulose, the glucose-derived downstream products such as sorbitol and mannitol are generated. Sorbitol and mannitol are produced by the hydrogenation of glucose that are used not only as sweeteners but also as precursors to isosorbide, lactic acid, 1,4-sorbitan, and other useful chemical compounds (Huber et al., 2003; Davda and Dumesic, 2004). It is first reported that supported metal catalysts can work on cellulose conversion into sorbitol and mannitol by a green environmental process, in the presence of Pt/γ-Al_2_O_3_, 31% yield of sugar alcohols (sorbitol: 25%, mannitol: 6%) were obtained in water solvent under 5 MPa H_2_ atmosphere at 463 K after 24 h (Fukuoka and Dhepe, 2006). Among the supported metal catalysts, Pt and Ru catalysts gave the higher yields of the sorbitol and mannitol, while Ir, Pd, and Ni catalysts showed lower activity. The reaction mechanism suggested that H_2_ is adsorbed on the Pt surface dissociatively and the hydrogen species spill over onto the γ-Al_2_O_3_ surface reversibly. Hence, the acid sites for the hydrolysis of cellulose are not only due to the acidic surface sites intrinsic in the γ-Al_2_O_3_ but also generated *in situ* from H_2_. The acidic sites play a decisive role in the first of cellulose hydrolysis to glucose, and then the sorbitol formed by means of reducing the C=O group in glucose with Pt and H_2_. Currently, bifunctional catalysts which contain acid sites and metal sites show excellent activity on direct conversion of cellulose into sorbitol have become the focus of researchers (Han and Lee, 2012; Zhu et al., 2014; Romero et al., 2016, 2017). A bifunctional catalyst Ru/CCD-SO_3_H (sulfonic acid-functionalized carbonized cassava dregs supported ruthenium) was prepared and successfully employed for the hydrolysis of cellulose to sorbitol in a neutral aqueous solution (Li Z. et al., 2019). More than double yield of sorbitol 63.8% can be achieved under the optimal conditions at lower temperature 180°C and shorter time 10 h compared with Pt/γ-Al_2_O_3_ catalyst abovementioned. Meanwhile, there exists a strong synergistic effect between -SO_3_H and Ru nanoparticles, acid sites are necessary in the depolymerization of cellulose to glucose and the hydrogenation sites of Ru could promote the formation of sorbitol from glucose. Furthermore, except supported metal catalysts and bifunctional catalysts, emerging catalysts like Ru-Ni bimetallic catalysts (supported by mesoporous carbon, activated carbon, and carbon nanotubes) also already show their potential for enhance one-pot hydrolytic hydrogenation of cellulose to sugar alcohols (Alonso et al., 2012; Pang et al., 2012; Sankar et al., 2012; Ribeiro et al., 2017). The result reveals that there is an interaction between both metals, and the presence of Ni improved the catalytic performance of the Ru monometallic catalysts both in terms of activity and selectivity. Furthermore, the yield of sorbitol is over 70% in a very short time (1 h) if the catalyst and cellulose are ball milled together. In addition, mannose can be obtained from glucose epimerization in the presence of acid through an intramolecular carbon shift as the Bilik reaction claimed. Another possible way to form mannose is from a reverse 1,2-hydride transfer from fructose, and further hydrogenation leads to the formation of mannitol (Gunther et al., 2012). When sodium tetraborate is incorporated into the active site of Sn-Beta zeolite, the selective epimerization of glucose to products would be promoted in aqueous media. The reaction proceeds by means of a 1,2 carbon shift of aldose wherein the bond is cleaved between C-2 and C-3 and a corresponding new bond is formed between C-1 and C-3, resulting in an inverted configuration by moving C-1 to the C-2 position.

Concisely, saccharides and sugar alcohols have always been considered as the link between the preceding cellulose and following platform chemicals; therefore, finding more efficient ways to prepare saccharides and sugar alcohols in aqueous phase has great significance for the utilization of cellulose.

**Scheme 2:**
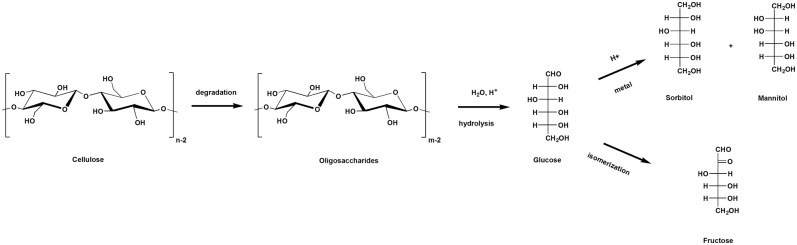
Conversion of cellulose to saccharides and sugar alcohols.

Scheme 2 shows the pathway of cellulose conversion to saccharides and sugar alcohols.

### Selective Hydrogenolysis of Glucose to Small Molecular Alcohols

Various small molecular alcohols have been widely used as solvents, fuel additives, and chemical synthesis intermediate; hence, efficient preparation of small molecular alcohols with ingenious catalysts design from direct hydrolysis and hydrogenolysis of cellulose has become the goal pursued by researchers.

#### Ethylene Glycol

In an earlier pioneering report, the process of EG production from cellulose in one-pot is significantly promoted by using a nickel-modified tungsten carbide catalyst (Ni-W_2_C/AC) instead of precious metal (Pt, Ru) catalysts, and the yield of EG was remarkably increased to 61% due to a synergistic effect between nickel and W_2_C (Degirmenci et al., 2011). W_2_C is mainly responsible for converting glucose into EG, and the addition of Ni could not only promote the formation of tungsten carbides but also limit the further hydrogenolysis of glucose to other polyols, thereby promoting the improvement of EG yield. After that, a battery of tungsten-based catalysts such as Ni-WO_3_/SBA-15, Ru/WO_3_, Raney Ni+H_2_WO_4_, W_X_C/MC, and Ru/AC+H_2_WO_4_ has been developed to promote the direct production of EG from cellulose with high activity and selectivity (Tai et al., 2013; Wang and Zhang, 2013; Cao et al., 2014; Li N. et al., 2017). The above literature reports a unified reaction mechanism: in the first, water-soluble oligosaccharides and glucose are obtained after the hydrolysis of cellulose in the presence of acid; then, glycolaldehyde is formed with catalysis of tungsten species by fracturing the corresponding C-C bond of oligosaccharides and glucose *via* retro-aldol condensation; finally, glycolaldehyde is catalyzed by a metal catalyst with hydrogenation characteristics to finish the production of target EG.

#### Ethanol

Besides an ideal additive to gasoline, biomass-derived ethanol can serve not only as a versatile precursor for the production of various chemicals but also as a green solvent that has received considerable attention (Farrell et al., 2006; Subramani and Gangwal, 2008). Traditionally, bio-ethanol is produced *via* the fermentation and distillation processes of pretreated lignocellulosic materials using enzymes and homogeneous acid (Fujita et al., 2004; Himmel et al., 2007; Binder and Raines, 2010). However, the enzymatic process is obviously limited in terms of many technological bottlenecks and economic challenges. For example, because the fermentation generally follows a reaction mechanism including the formation of a pyruvate intermediate and its subsequent decarboxylation, one mole of CO_2_ would be simultaneously released when one mole of pyruvate is converted into ethanol, thereby reducing the carbon atom efficiency and resulting in the theoretical yield of bio-ethanol confined to 67 mol% (Kennes et al., 2016). In contrast, the conversion of cellulose to ethanol by a chemical approach remains challenging due to a series of inherently complex reactions including hydrolysis, retro-aldol condensation, and hydrogenation are involved and some other side reactions may be caused by unstable intermediates. Therefore, the providential design of multifunctional and robust catalysts that can regulate all kinds of complex reactions efficiently is paramount. As reported, tungsten-based catalysts and Pt-Cu/SiO_2_ single-atom alloy catalyst were successfully applied to the two-step conversion of cellulose to ethanol, methyl glycolate(MG) is easily formed from glycolaldehyde intermediate when the reactions were carried out in methanol solvent, and further hydrogenation is necessary to obtain ethanol after MG hydrogenated to EG (Xu et al., 2017; Yang et al., 2018). Noting that the EG is the precursor of ethanol production and could get a high yield in deionized water which avoids the use of organic solvent, thereby the process that conversion of cellulose to ethanol is thought highly of in aqueous phase.

**Scheme 3:**
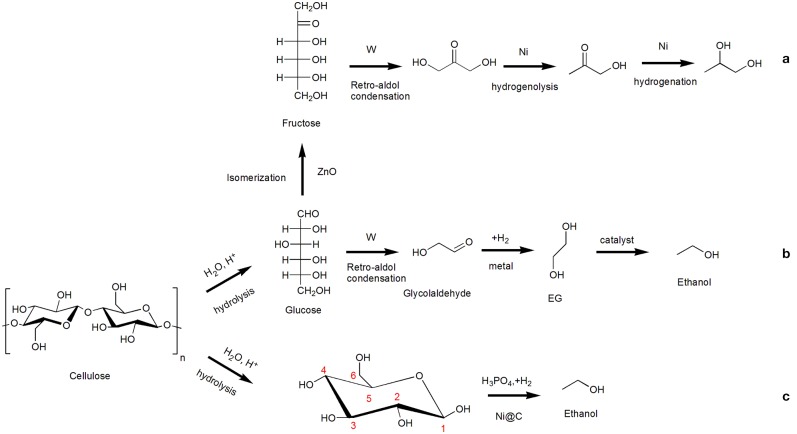
Conversion of cellulose to C2/C3 alcohols.

Lately, the study about one-pot conversion of cellulose into ethanol catalyzed by a combination of tungstic acid and zirconia-supported Pt nanoparticles (H_2_WO_4_-Pt/ZrO_2_) was presented (Song et al., 2019). As the studies revealed, firstly, *via* the retro-aldol condensation, H_2_WO_4_ is deemed to be responsible for the breakage of the C-C bond in glucose unit, particularly the C2-C3 bond. Secondly, Pt/ZrO_2_ mainly works on the hydrogenation of the C=O bond in glycolaldehyde into EG and the further hydrogenolysis of the C-OH bond in C2 intermediates into ethanol. Lastly, appropriate fractions of Pt^0^ and Pt^2+^ on ZrO_2_ support impose restrictions on over-hydrogenolysis which are crucial to promote the formation of ethanol. An ethanol yield of 32% and EG yield of 24% at the same time were obtained from cellulose at 523 K in 5 h under 4 MPa H_2_ atmosphere with deionized water. Later, a multifunctional Ru-WOx/HZSM-5 catalyst was designed appropriately for one-pot efficient transformation of cellulose to ethanol *via* a series of cascade reactions (Li C. et al., 2019). Characterizations revealed that Ru and WOx nanoparticles were highly dispersed on the surface of HZSM-5 and formed Ru_3_W_17_ alloy, which displayed a synergistic catalytic effect along with moderate acidic sites: moderate acid sites are responsible for cellulose hydrolysis, glucose retro-aldol condensation, and EG dehydration, and Ru_3_W_17_ alloy sites are responsible for EG hydrogenation to ethanol. Meanwhile, the key retro-aldol condensation reaction could be promoted with the addition of Ru/WOx by suppressing oligomerization led to an increase in the yield of ethanol from 76.8% to 87.5 over 1 wt% cellulose under mild conditions in water. When taking the advantages of Pt/WOx into consideration as the parent catalyst on account of its high efficiency in the selective cleavage of the secondary C-O bond of glycerol to form 1,3-propanediol (Wang et al., 2016; Zhao et al., 2017), another multifunctional catalyst Mo/Pt/WOx was successfully designed to adapt to tandem reactions for one-pot production of ethanol from cellulose (Yang et al., 2019). As expected, Pt/WOx shows a superior activity and selectivity in the critical process of EG to ethanol by the introduction of extra certain content Mo, and the activity and selectivity effected significantly with different sedimentary sequence of Mo and Pt. Finally, constituent content determined catalyst 0.1Mo/2Pt/WOx displayed an idiosyncratic ability for cellulose conversion to ethanol, and experimentally verified that the EG hydrogenolysis to ethanol by breaking the C-O bond is the rate-determining step in the whole tandem catalysis reaction. Similarly, the reaction pathways are consistent with previous reports: EG as a key intermediate can be obtained in the presence of Pt from glycolaldehyde (generated after cellulose hydrolysis under the catalysis of WO_X_), and further production of ethanol from EG hydrogenolysis would be proceeded over Mo/Pt/WOx catalyst. Summarizing the above three articles on the direct preparation of ethanol from cellulose, we can know that they have the common point of converting cellulose to the intermediate glycolaldehyde by a tungsten-based catalysts, and then make the conversion of glycolaldehyde to EG under a noble metal with hydrogenation effect, finally, the end product ethanol could be obtained by EG hydrogenolysis, as shown in Scheme 3b.

Distinctively, one-pot production of ethanol from cellulose hydrogenolysis using chemo-catalysts Ni@C accompanied by the addition of phosphoric acid (H_3_PO_4_) in water was reported which demonstrated a different reaction mechanism compared with the conversion path of EG in essence (Liu et al., 2019). As revealed by the experimental results, H_3_PO_4_ plays the dual roles as acid not only in catalyzing hydrolysis of cellulose to glucose but also in coordinating with glucose to form cyclic di-ester structure (formed *via* dehydration of C-OHs in glucose and P-OHs in H_3_PO_4_) for activating the corresponding C-C and C-O bonds for further production of ethanol. A variety of catalyst characterizations revealed that Ni was encapsulated by graphene layers, and noted that the fewer the number covering graphene layers, the better the catalytic performance; furthermore, the delocalized π electrons of graphene layers weaken absorption of H atoms on Ni@C surface by creating the electronic negative surface which could facilitate the direct conversion of cellulose to ethanol. Significantly, the result of ^13^C NMR analysis shows that the generation of ethanol from glucose by the cleavage of C2-C3 and C4-C5 bond as well as the hydroxyl group is removed from the C1 and C6 site in glucose, as shown in Scheme 3c. Ni@C catalyst also shows high stability and activity for at least eight runs under optimal conditions (above 60% yield of ethanol at 200°C in 3 h with 40 ml of 0.06 M H_3_PO_4_ aqueous solution under H_2_ atmosphere). In particular, higher ethanol yield (69.1%) in Ni@C-H_3_PO_4_ catalytic system than theoretical value by glucose fermentation (66.7%) and the prevention of using noble metals provide an opportunity for practical and economical application of direct conversion of cellulose to ethanol. Table 2 shows the summary of one-pot conversion of cellulose to ethanol.

**Table 2:** The summary work of one-pot conversion of cellulose to ethanol.

**Entry**	**Catalyst**	**Cellulose dosage(g)**	**Solvent**	**Temperature (K)**	**Time (h)**	**Ethanol yield (%)**	**References**
1	0.15 g H_2_WO_4_-Pt/ZrO_2_	0.2	H_2_O	523	5	32	Song et al., 2019
2	0.1 g Ru-WOx/HZSM-5	0.1	H_2_O	508	10	59	Li C. et al., 2019
3	0.1 g Mo/Pt/WOx	0.15	H_2_O	518	2	43.2	Yang et al., 2019
4	0.15 g Ni@C	0.4	H_3_PO_4_	473	3	69.1	Liu et al., 2019

#### 1,2-Propylene Glycol

In order to increase the yield of 1,2-propylene glycol(1,2-PG) in the process of cellulose conversion, multifunctional catalyst (Ni-W-ZnO/β) was tailored by supporting nickel and tungsten on β-zeolite with addition of ZnO (Gu et al., 2019). Each component of Ni-W-ZnO/β catalyst has its specific role shown below. Due to the abundant acid sites, β-zeolite exhibited a high catalytic performance on the hydrolysis of cellulose to glucose. Further isomerization of glucose to fructose was promoted by addition of ZnO, which is a key step to generate fructose precursor. Ni is effective in the hydrogenation of dihydroxyacetone to acetol and acetol to target 1,2-PG while W facilitates bond cleavage *via* a retro-aldol condensation of fructose to dihydroxyacetone intermediate. As shown in Scheme 3a, Ni-W-ZnO/β catalyst shows a high activity and selectivity in the conversion of cellulose to C2/C3 glycols (1,2-PG specially), total yield of EG and 1,2-PG reached 70.1% with 1,2-PG accounting for 51.1%.

Knowing from the above process, hydrogenolysis is the decisive step in the formation of small molecular alcohols, which results in the cleavage of corresponding C-C and C-O bonds by hydrogen and also be regarded as a promising available technology for future biorefinery concepts. Furthermore, how to achieve efficient hydrogenolysis and the exploration of hydrogenolysis mechanism should be focused on in the future research.

### Production of 5-Hydroxymethylfurfural and Its Derivatives

#### 5-Hydroxymethylfurfural

**Scheme 4:**
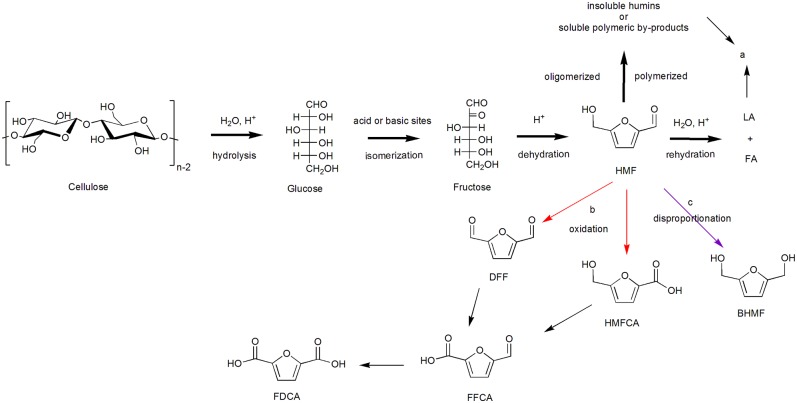
Production of 5-hydroxymethylfurfural (HMF) and its derivatives [mainly 2,5-furandicarboxylic acid (FDCA)].

HMF has always been considered as a bridge between renewable biomass resources and platform compounds for its versatile function in terms of obtaining medical drugs, fuel additives, and other bulk chemicals (Körner et al., 2019). It was first reported in 1951 by Newth on the synthesis of HMF from carbohydrates (Newth, 1951), and publications on HMF chemistry have increased significantly in recent years. In the beginning, homogeneous catalysts were favored in the dehydration of fructose to HMF, like HCl (Kuster and Temmink, 1977), oxalic acid (Elhall et al., 1983), H_2_SO_4_ (Antal et al., 1990), and H_3_PO_4_ (Tarabanko et al., 2006). With homogeneous catalysts, in order to obtain higher HMF yields, harsh reaction conditions containing higher temperatures and higher pressures are generally required, 50% HMF yields and 95% conversion of fructose could be obtained in the presence of H_2_SO_4_ under 250°C (Antal et al., 1990). Although the homogeneous catalysts can obtain a certain higher HMF yield, there still exists the problems about difficult separation of homogeneous acid catalysts from the solvent after reaction and the corrosive to the reaction equipment; moreover, the selectivity of the reaction is rather low. Later, related work on HMF synthesis from fructose was published using heterogeneous catalysts in aqueous environment by Carlini et al. When niobium phosphate-based catalysts were applied, quite high selectivity (over 85%) of HMF could be obtained, but only about 30% conversion of fructose were observed in the meantime (Carlini et al., 1999). Cubic zirconium pyrophosphate catalyst (Benvenuti et al., 2000) and vanadyl phosphate catalysts (Carlini et al., 2004) also showed similar reaction patterns, a higher HMF selectivity corresponds to a lower fructose conversion. Generally, large amounts of HMF are unstable in water, its further rehydration to levulinic acid (LA) and formic acid (FA) can be facilitated under acid conditions. More than that, HMF may also be oligomerized or polymerized by itself or with fructose to generate insoluble humins or soluble polymeric by-products, lead to the decrease of HMF yield, shown as Scheme 4a. As a comparative experiment on γ-TiP catalyst has shown, HMF was extracted with an organic solvent—methyl isobutyl ketone (MIBK) when the reaction was halfway through and at the end, the yield of HMF increased apparently from 39 to 67%, although the reaction time was already shortened from 2 to 1 h (Benvenuti et al., 2000). Due to the timely separation of generated HMF from the water solvent, the occurrence of side reactions is reduced, thereby greatly increasing the HMF yield.

When the substrate turns to glucose, the formation of HMF become more complex and difficult attributed to the requirements of Lewis acid for the isomerization of glucose to fructose firstly. In the process of HMF production, Lewis acid sites are prerequisite for the isomerization of glucose into fructose; the lack of Lewis acid sites could make the isomerization rate go down, but excessive Lewis acid sites will result in the formation of undesired by-products and humins, further reduce the HMF selectivity; similarly, Brønsted acid sites are active for dehydration of fructose, the lack of Brønsted acid sites cannot make a full dehydration of fructose to HMF, limiting the generation of HMF, while excessive Brønsted acid sites may suppress the isomerization reaction (Zhang M. et al., 2015; Kreissl et al., 2017; Li X. et al., 2017). Therefore, in order to achieve the cooperative catalytic action for the conversion of glucose to HMF, it is extremely important to find a balance between Lewis acid sites and Brønsted acid sites. In addition to acidic sites, water tolerance is the intrinsic requirement for catalyst when converting glucose to HMF in aqueous phase, thereby niobium-based catalysts have attracted the attention of researchers due to their good water-tolerant property and tunable surface acid density. The acid properties of Lewis acid sites and Brønsted acid sites could be adjusted by controlling the synthesis pH values of porous niobium phosphate catalyst. When NbPO_4_ catalyst was synthesized at pH = 7, the amounts of Lewis acid and Brønsted acid were balanced and highest 33.6% yield of HMF could be obtained from glucose under optimal reaction conditions in pure water (Zhang M. et al., 2015). According to the literature (Watanabe et al., 2005a,b; Chareonlimkun et al., 2010), more than Lewis acidic sites, basic sites present on metal oxides can also lead to the isomerization of glucose to fructose. Bifunctional catalyst ZrO_2_-TiO_2_ possesses both basic sites for isomerization and acid sites for dehydration achieved a relatively high HMF yield of 29% in aqueous phase. The result of TPD analysis indicated that ZrO_2_ mainly works as an isomerization catalyst to form fructose from glucose, and TiO_2_ served as an acidic catalyst mainly responsible for the further dehydration of fructose to HMF.

**Scheme 5:**
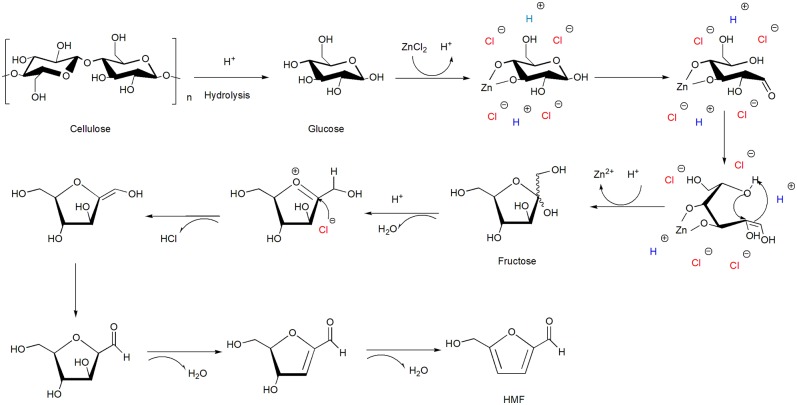
Schematic diagram of 5-hydroxymethylfurfural (HMF) formation from cellulose in the presence of ZnCl_2_.

Direct conversion of cellulose to HMF is more challenging than that from fructose and glucose, since the dissolution of cellulose is required at first in aqueous phase. Therefore, it will be meaningful to find an aqueous solution which could not only hydrolyze cellulose but also promote HMF production. Fortunately, it is reported that cellulose could be dissolved in aqueous ZnCl_2_ solution with a concentration ≥60 wt% by forming a Zn–cellulose complex (Cao et al., 1994, 1995). Moreover, the aqueous ZnCl_2_ solution also exhibited strong acidity which would be able to facilitate the hydrolysis of cellulose and the dehydration of fructose to HMF, as shown in Scheme 5. The Zn^2+^ ions in the concentrated aqueous ZnCl_2_ solutions can be combined with two adjacent hydroxyl groups on glucose, which may play a same key role like Cr^2+^ in the isomerization of glucose to fructose (Xu and Chen, 1999; Pidko et al., 2010). As high as 30.4% HMF yield can be directly obtained from cellulose in 63 wt% high concentrated ZnCl_2_ solution (Deng et al., 2012). In a low concentrated solution, since the coordination between H_2_O and Zn^2+^ ions is stronger than that between hydroxyl groups and Zn^2+^ ions, there is no excess Zn^2+^ to combine with hydroxyl group, further lead to a lower HMF yield. In short, although aqueous solution achieved a lower yield than organic solvents or ionic liquids in the production of HMF from fructose, glucose, and cellulose, aqueous-phase reaction system has the most available and economic value. Hence, as to researchers, it is very meaningful to seek out novel catalysts to improve both yield and selectivity as far as possible in the conversion of biomass carbohydrates to HMF.

Table 3 shows an overview of published work about HMF production under aqueous conditions.

**Table 3:** Production of 5-hydroxymethylfurfural (HMF) under aqueous conditions.

**Entry**	**Substrate**	**Catalyst**	**Temperature (^**°**^C)**	**Time**	**Yield (%)**	**Conversion (%)**	**Selectivity (%)**	**References**
1	Fructose	HCl	90	7 h	43	72	60	Kreissl et al., 2017
2	Fructose	Formic acid	175	45 min	56	56	100	Li X. et al., 2017
3	Fructose	γ-Tip	100	2 h	39	57	69	Benvenuti et al., 2000
4	Fructose	FeVOP	80	1 h	60	71	84	Carlini et al., 2004
5	Glucose	DyCl_3_	140	2 h	12	30	40	Seri et al., 2001
6	Glucose	TiO_2_-ZrO_2_	250	5 min	29	44	67	Chareonlimkun et al., 2010
7	Glucose	MnPO_4_	160	90 min	18	72	25	Xu et al., 2018
8	Glucose	NbPO_4_	140	60 min	33.6	68.1	49.3	Zhang et al., 2015b
9	Cellulose	ZnCl_2_+HCl	120	–	30.4	–	–	Deng et al., 2012
10	Cellulose	Bimodal-HZ-5	190	4 h	46	67	69	Li and Yu, 2014

#### 2,5-Furandicarboxylic Acid

FDCA, obtained after HMF oxidation, is an important monomer in polymer and chemical industry, which can replace petroleum-based terephthalic and isophthalic acids to reduce our dependence on traditional petroleum (Papageorgiou et al., 2016). There are general three intermediate products in the production of FDCA from HMF oxidation, 5-hydroxymethyl-2-furancarboxylic acid (HFCA), 2,5-diformylfuran (DFF), and 5-formyl-2- furancarboxylic acid (FFCA), as shown in Scheme 4b. When the aldehyde group on HMF is first selectively oxidized to a carboxyl group, intermediate product HMFCA is obtained. On the contrary, when the hydroxyl group on HMF is first selectively oxidized to an aldehyde group, intermediate product DFF can be obtained. After that, under oxygen conditions, an aldehyde group on DFF can be converted to carboxyl group, and hydroxyl group on HMFCA is oxidized to aldehyde group, both of them could form the other intermediate product 5-formyl-2- furancarboxylic acid (FFCA). FDCA can be obtained after further oxidation of FFCA. Noble metals (like Pt, Pd, and Au) supported by carbon or metal oxides (like Al_2_O_3_, TiO_2_, and CeO_2_) have been found to be efficient for the oxidation of HMF to FDCA (Davis et al., 2011; Saha et al., 2013; Li Q. et al., 2019). Several supported Pt, Pd, and Au metal catalyst reactivities were investigated for the oxidation of HMF to FDCA under identical conditions in the aqueous phase of sodium hydroxide (Davis et al., 2011). Pt/C, Pd/C, Au/C, and Au/TiO_2_ catalysts were synthesized and applied. In the presence of Pt/C and Pd/C, higher yield of FDCA could be achieved, while mainly intermediate product HMFCA were formed did not continue to be converted to FDCA in the presence of Au/C and Au/TiO_2_ under identical conditions (690 kPa O_2_, 295 K, 6 h, and 0.3 M NaOH). With the operation of increasing O_2_ pressure to 2,000 kPa, reaction time to 22 h, and NaOH concentration to 2.0 M, higher 72% and 80% yield of FDCA can be achieved over Au/C and Au/TiO_2_ respectively. The experimental result indicates that Pt and Pd can activate the hydroxyl group of HFCA under a mild condition and lead to the formation of FDCA, whereas Au required higher O_2_ pressure and NaOH concentration. The reason can be revealed as the literature reported that a large amount of OH^−^ is required to activate the hydroxyl group to form aldehyde group intermediate, which can subsequently be oxidized to carboxyl group in the presence of Au catalyst and O_2_ (Ketchie et al., 2007). Noting that in the presence of NaOH, undesirable by-products such as 2,5-bis(hydroxymethyl)furan (BHMF) may be formed by disproportionation reaction of HMF. Scheme 4c.

**Scheme 6:**
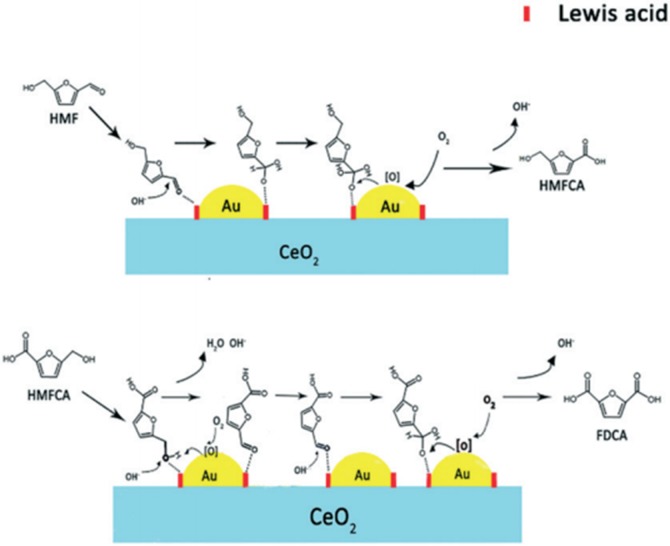
Possible reaction mechanism for oxidation of 5-hydroxymethylfurfural (HMF) to 2,5-furandicarboxylic acid (FDCA). Reprinted with permission from Li C. et al. (2019). Copyright 2019 Royal Society of Chemistry.

According to the previous work in our group, different morphologies of CeO_2_ support Au catalysts (Au/CeO_2_-rod, Au/CeO_2_-cube and Au/CeO_2_-oct) synthesized *via* hydrothermal method and deposition–precipitation exhibited high selectivity of HMF oxidation to FDCA (Li Q. et al., 2019). Combining with experimental results and catalyst characterization, valence of Au and the interfacial acidic properties can be affected by the oxygen vacancies on Au-CeO_2_ interface, which determined the catalytic activity. The Au/CeO_2_-rod catalysts with the highest oxygen vacancies achieved higher yield of FDCA than Au/CeO_2_-cube and Au/CeO_2_-oct catalysts in aqueous solution of sodium hydroxide. Further characterization of catalysts shows that Au/CeO_2_-rod possesses more interfacial Lewis acidic sites and cationic Au between Au nanoparticles and CeO_2_-rod. Hence, we proposed a mechanism of synergistic effect between the interfacial Lewis acid sites, OH^−^, and neighboring Au particles, by which the groups (hydroxyl, aldehyde) and molecular O_2_ can be activated efficiently, thereby promoting the progress of oxidation reaction, as shown in Scheme 6. And Table 4 exhibits the experimental results about production of FDCA from HMF according to references (Davis et al., 2011; Li Q. et al., 2019).

**Table 4:** Davis et al. (2011) and Li Q. et al. (2019) experimental results about production of 2,5-furandicarboxylic acid (FDCA) from 5-hydroxymethylfurfural (HMF) in aqueous phase.

**Entry**	**Catalyst**	**Temperature (**°**C)**	**Time(h)**	**Yield (%)**	**Conversion (%)**	**Selectivity (%)**
1^a^	Pt/C	22	6	79	100	79
2^a^	Pd/C	22	6	71	100	71
3^a^	Au/C (WGC)	22	6	8	100	8
4^a^	Au/C(sol)	22	6	7	100	7
5^a^	Au/TiO_2_	22	6	8	100	8
6^b^	Au/C(sol)	22	6	31	100	31
7^b^	Au/TiO_2_	22	6	32	100	32
8^b^	Au/C(sol)	22	22	72	100	72
9^b^	Au/TiO_2_	22	22	80	100	80
10^c^	Au/CeO_2_-rod	130	2.5	87.4	100	87.4
11^c^	Au/CeO_2_-cube	130	2.5	19	100	19
12^c^	Au/CeO_2_-otc	130	2.5	2	100	2

a *Reaction conditions: 0.15 M HMF solution in 0.3 M NaOH, metal:HMF = 6.67 × 10^−3^ mol/mol, P = 690 kPa O_2_*.

b *Reaction conditions: 0.1 M HMF solution in 2.0 M NaOH, metal:HMF = 8.0 × 10^−3^ mol/mol, P = 2,000 kPa O_2_*.

c *Reaction conditions: 2.5 h, 130°C, 0.5 MPa O_2_, molar ratio of NaOH/HMF = 4, molar ratio of HMF/Au = 400, 0.5 mmol HMF, 20 ml H_2_O*.

Although with the addition of mineral base, noble metal catalysts show excellent performance and high yields of FDCA can be achieved, the high costs and produced wastewater may limit their practical application. Therefore, the exploitation of non-noble metal catalysts and greener reaction medium without mineral base or acid should be investigated majorly in the synthesis of FDCA.

### Production of Liquild Alkanes *via* Hydrogenolysis and Hydrodeoxygenation

Direct hydrodeoxygenation of cellulose would lead to the formation of gasoline alkanes, mainly pentanes and hexanes. Taking the insoluble property and complex structure of cellulose into consideration, cellulose needs to be first hydrolyzed to soluble hexitol, then pentanes and hexanes would be formed *via* consecutive dehydration, hydrogenation, or hydrogenolysis. Numerous research efforts on hydrogenolysis and hydrodeoxygenation of cellulose to gasoline alkanes have been made (Chen et al., 2013; Liu C. et al., 2014; Liu S. et al., 2014; Beeck et al., 2015; Liu et al., 2015; Venkatakrishnan et al., 2015; Xi et al., 2016).

**Scheme 7:**
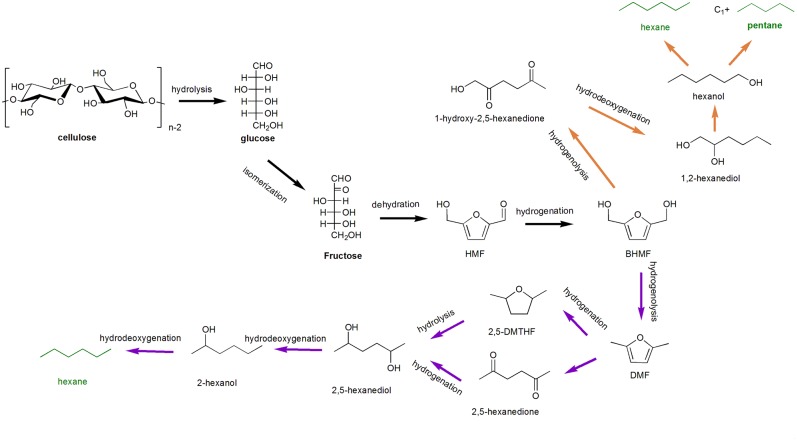
Probable pathway of cellulose conversion to pentane and hexane *via* 5-hydroxymethylfurfural (HMF).

As shown in Scheme 7, reaction pathway *via* HMF: the aldehyde group on HMF is reduced to hydroxyl group leads to the formation of BHMF, and two kinds of intermediate precursor 1-hexanol and 2-hexanol could be obtained. 1-hexanol conversion pathway can be expounded as the following: in the presence of metal catalysts and acid conditions, 1-hexanol can be directly converted to hexane by dehydration and hydrogenation, or fracture of C-O bond under the action of metal sites; on the other hand, pentane could be generated from hexanol *via* the cleavage of C1-C2, meanwhile along with the formation of one molecule CO_2_ or methane. Both pentane (mainly) and hexane could be obtained. However, for the conversion of 2-hexanol, reactions are prone to dehydration and hydrogenation of -OH than hydrogenolysis, lead to the generation of hexane. Noting that the conversion route *via* HMF mainly occurs in a reaction solvent containing an organic phase (n-decane, n-dodecane, etc.) or subcritical water (Osaka et al., 2013). There are also some researches on the conversion of cellulose to pentane and hexane in water-organic biphasic system (Chen et al., 2013; Liu S. et al., 2014; Beeck et al., 2015). Therefore, here we mainly introduce some representative transformation of pentanes and hexanes in aqueous phase.

**Scheme 8:**
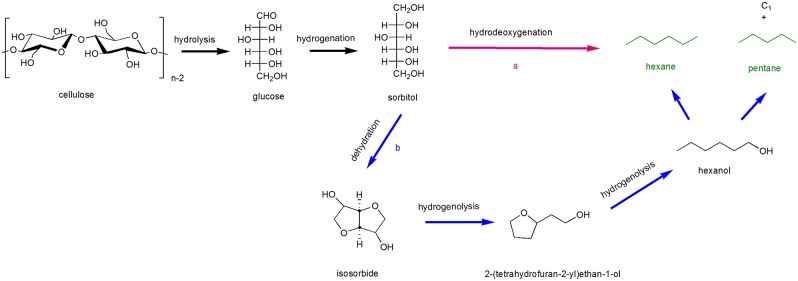
Probable pathway of cellulose conversion to pentane and hexane *via* sorbitol.

There are generally two pathways of pentanes and hexanes formation in aqueous phase that are proposed from intermediate precursors sorbitol (Chen et al., 2013). As shown in Scheme 8a, sorbitol can be obtained from the hydrogenation of glucose after cellulose hydrolysis, which can be directly converted to hexane *via* hydrodeoxygenation under metal-acid catalytic systems; on the other hand, Scheme 8b, after dehydration of sorbitol to isosorbide, 1-hexanol can be achieved *via* further hydrogenolysis of isosorbide, pentanes and hexanes(mainly) could also be obtained under metal-acid catalytic systems by fracturing the corresponding C-O and C-C band (Li and Huber, 2010).

**Scheme 9:**
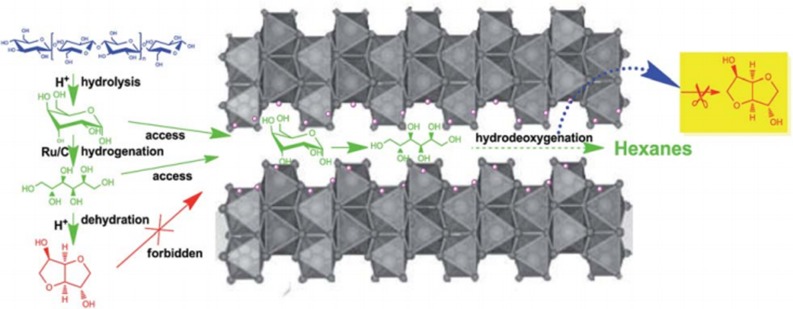
Conversion of cellulose to hexanes over LiNbMoO_6_ and Ru/C in aqueous phosphoric acid. Reprinted with permission from Liu C. et al. (2014). Copyright 2014 Royal Society of Chemistry.

It is reported that layered LiNbMoO_6_ catalysts combined with Ru/C catalyst are conducive to the preparation of liquid alkanes from direct cellulose conversion in low concentration phosphoric acid aqueous solution (Liu S. et al., 2014). In this process, glucose that was obtained after cellulose hydrolysis could be hydrogenated to sorbitol in the presence of Ru/C catalyst, and the formation of isosorbide from sorbitol was inhibited significantly with the addition of layered LiNbMoO_6_ catalysts, so that the formation of hexane is promoted efficiently *via* hydrodeoxygenation of sorbitol by combining LiNbMoO_6_ catalysts and Ru/C catalyst. According to the analysis results of catalysts and experiments, glucose and sorbitol, not isosorbide, are allowed to enter the interlayer pores due to the steric hindrance of the pores between the layered LiNbMoO_6_ catalyst layers, so the process of converting sorbitol into isosorbide is inhibited, and this mechanism is conducive to the formation of hexane, as shown in Scheme 9. Benefit by the unique layer structure of LiNbMoO_6_ and property of Ru/C, high yield of hexanes (72% C) and pentane (5.9% C) could be obtained directly in low-concentration phosphoric acid aqueous solution by one-pot conversion of cellulose *via* hydrolysis and hydrodeoxygenation.

When isosorbide is formed after the dehydration of sorbitol, the reaction pathway to pentane and hexane production is obviously different from abovementioned, which is hardly converted to alkanes by further hydrodeoxygenation. Pt/NbOPO_4_ multifunctional catalyst was investigated for alkane production from sorbitol in aqueous solution (Xi et al., 2016), the final product of sorbitol dehydration is isosorbide, also an important intermediate in this process. With following ring opening hydrogenation reactions of isosorbide and two successive hydrogenolysis steps lead to the formation of hexanol, further converted to hexane and pentane *via* C-O band cleavage. In order to have a better understanding of the conversion process, the activation energy of isosorbide hydrogenolysis and sorbitol dehydration was calculated, that is, 72.7 and 147.6 kJ/mol, which are much lower compared to Pt/H-Beta (92 and 171.3 kJ/mol) catalysts. It was also revealed that isosorbide hydrogenolysis is the rate-determining step. Moreover, the strong and high acid amount on NbOPO_4_ supporter and the promoter effect of component NbO_x_ on C-O bond cleavage are responsible for sorbitol dehydration and isosorbide hydrogenolysis, respectively. With good catalytic performance of Pt/NbOPO_4_ catalyst, 55.9% yield of hexane and 4.8% yield of pentane can be achieved from sorbitol under optimal reaction conditions in water phase.

Overall, noble metal catalysts show an efficient and stable catalytic reaction system in the conversion of cellulose to produce alkanes. However, noble metal catalysts cannot be widely used due to its limited availability on earth, which motivated the exploration of alternative non-noble metal catalysts. Ni/HZSM-5 catalyst was investigated by aqueous-phase reforming of sorbitol to bio-gasoline (Zhang et al., 2012). Total 36.4% yield of C5-C6 alkanes can be achieved at 240°C under 4 MPa H_2_. By comparing the characterization analysis results over H_2_-TPR/TPD of calcined catalyst at different temperatures (400, 500, 600, and 700°C), the Cat-500 catalyst has the largest H_2_ consumption of 1.34 mmol g^−1^ and higher hydrogen desorption, which indicated that decomposed NiO species were reduced completely accompany with the appearance of more Ni active sites on HZSM-5. In addition, the catalyst in lower calcination temperature is not able to provide enough active sites while Ni particles can easily be sintered in higher calcination temperature. Moreover, according to N_2_ physical adsorption results, Ni/HZSM-5 catalyst, calcined at 500°C, possess a higher surface area and appropriate amount of macropores. On the one hand, higher surface area means more active sites that could be beneficial to reactions; on the other hand, appropriate amount of macropores could promote the desorption of the target products, so the side reactions (for example, the cleavage of alkanes) could also be suppressed, thereby further enhancing the yield and selectivity of alkanes.

Table 5 shows the summary work about pentane and hexane preparation *via* different catalysts.

**Table 5:** Summary work about hexanes and pentanes preparation under different catalytic systems in aqueous phase.

**Entry**	**Catalysts**	**Solvent**	**Carbon balance (%)**	**Yield (%)**			**References**
				**Hexane**	**Pentane**	**Others**	
1^a^	Ru/C	H_2_O	95.4	1.9	1.1	92.4	Liu S. et al., 2014
2^a^	Ru/C	H_3_PO_4_	93.6	23.3	12.4	57.9	Liu S. et al., 2014
3^a^	Ru/C+MCM-41	H_3_PO_4_	93.9	48.6	9.6	35.7	Liu S. et al., 2014
4^a^	Ru/C+HZSM-5	H_3_PO_4_	93.4	36.6	16.4	40.4	Liu S. et al., 2014
5^a^	Ru/C+γ-Al_2_O_3_	H_3_PO_4_	94.5	35.3	14.4	44.8	Liu S. et al., 2014
6^a^	Ru/C+SBA-15	H_3_PO_4_	95	29.7	13.8	51.5	Liu S. et al., 2014
7^a^	Ru/C+HNbMoO_6_	H_3_PO_4_	88.4	65.9	6.9	15.6	Liu S. et al., 2014
8^a^	Ru/C+LiNbMoO_6_	H_3_PO_4_	88.6	72	5.9	10.7	Liu S. et al., 2014
9^a^	Ru/C+LiNbWO_6_	H_3_PO_4_	84.5	47.7	5.5	31.3	Liu S. et al., 2014
10^b^	Pt/NbOPO_4_	H_2_O	>66	55.9	4.8	>5.3	Xi et al., 2016
11^b^	Pd/NbOPO_4_	H_2_O	>37.7	23.5	5.1	>9.1	Xi et al., 2016
12^b^	Ru/NbOPO_4_	H_2_O	>53.4	8.9	7.8	>36.7	Xi et al., 2016
13^b^	Ir/NbOPO_4_	H_2_O	>58.1	6.1	3.1	>48.9	Xi et al., 2016
14^b^	Rh/NbOPO_4_	H_2_O	>48.5	10.2	15.2	>23.1	Xi et al., 2016
15^c^	Ni/HZSM-5	H_2_O	35.3	18.3	4.7	12.3	Zhang et al., 2012
16^d^	Ni/HZSM-5	H_2_O	47.6	30	6.4	11.2	Zhang et al., 2012
16^e^	Ni/HZSM-5	H_2_O	45	23.7	8.0	13.3	Zhang et al., 2012

a *Reaction conditions: 0.8 g microcrystalline cellulose, 0.2 g Ru/C, 230°C, 40 ml solvent, 6 MPa H_2_, 24 h, others = C1-C4 alkanes, glucose, cellobiose, sorbitol, sorbitans, isosorbide, 1-hexanol, 1-pentanol, 1,6-hexanediol, ethylene glycol, and propylene glycol*.

b *Reaction conditions: 1 g sorbitol, 0.3 g catalysts, 250°C, 20 g H_2_O, 4 MPa H_2_, 12 h, others = isosorbide (mainly), sorbitan, 2-(tetrahydrofuran-2-yl)ethan-1-ol, and hexanol*.

c *Reaction conditions: 0.05 mol sorbitol, 3.0 g catalyst, 240°C, 150 ml deionized water, 3 MPa H_2_, others = C1-C4 alkanes*.

d *Reaction conditions: 0.05 mol sorbitol, 3.0 g catalyst, 240°C, 150 ml deionized water, 4 MPa H_2_, others = C1-C4 alkanes*.

e *Reaction conditions: 0.05 mol sorbitol, 3.0 g catalyst, 240°C, 150 ml deionized water, 5 MPa H_2_, others = C1-C4 alkanes*.

Though a series of multifunctional catalysts are investigated for the production of C5-C6 alkanes, the reaction routes and catalytic mechanism are not clearly enough. Accordingly, the design of catalysts for involved complicated reaction process (hydrolysis, dehydration, hydrogenation, and hydrogenolysis, etc.) should be more targeted and efficient.

## Enzymatic Conversion of Cellulosic Materials and the Production of Derived Green Solvents

As an effective and promising process of cellulose conversion, enzyme treatment methods are thought highly of due to its specificity to the corresponding substrate. Typically, a mixture of several enzymes, cellulase, consists of endo-1-4-β-glucanase, cellobiohydrolase, and β-glucosidase, works synergistically on cellulose hydrolysis process (Nigam, 2013). Firstly, endoglucanse cleaves β-1, 4–glycosidic linkage of D–glucan chains casually in the amorphous regions of cellulose or the surface of microfibrils, so that free chains that contain both reducing and non-reducing ends could be obtained. Then, cellobiose can be achieved while cellobiohydrolase acts on reducing and non-reducing ends. Ultimately, the cellobiose would be converted into glucose by β-glucosidase (Zabed et al., 2016). Moreover, lignocellulosic materials could also be converted by enzymes (Skiba et al., 2016). In the presence of CelloLux-A and BrewZyme BGX industrial enzymes, the hydrolysis of lignocellulosic materials from miscanthus and oat husks was studied. Several monosaccharides including xylose, mannose, glucose, and galactose were obtained after the substrate is treated with enzymes. Expectedly, glucose has the highest yield among those monosaccharides, 75.8 and 53.5% yields can be achieved from miscanthus and oat husks. Surprisingly, bioethanol, a derived green solvent, could be obtained in a high yield with further fermentation after enzymatic hydrolysis.

**Scheme 10:**
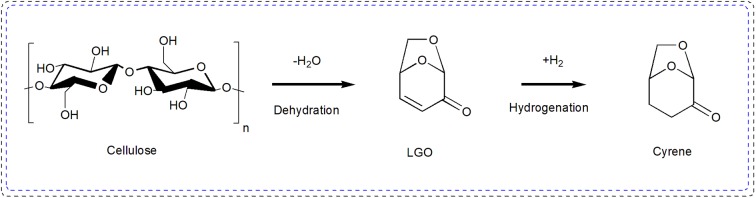
Synthetic route to levoglucosenone (LGO) and Cyrene from cellulose.

In addition to bioethanol, levoglucosenone (LGO) and dihydrolevoglucosenone (Cyrene) are also considered as green solvents and building blocks of platform chemicals. LGO is the precursor of synthetic Cyrene that was obtained after cellulose dehydration, which could be subsequently hydrogenated to Cyrene, as shown in Scheme 10. Cyrene has the potential in many applications, such as in the field of graphene manufacturing due to its optimal polarity and high viscosity (Salavagione et al., 2017); a replacement for toxic dipolar aprotic solvents, like DMF, NMP, and sulfolane (Camp, 2018); used to synthesize metal-organic framework (Zhang et al., 2016). However, the synthesis of Cyrene mostly used Pd-based catalysts with additional hydrogen. Distinctively, an alkene reductase (OYE 2.6) shows a high performance in the reduction of levoglucosenone to Cyrene (Mouterde et al., 2018), high levoglucosenone conversion of 99 and 99% yield of Cyrene can be achieved by continuous extraction. Furthermore, the formation of side product (1R,2S)-2-hydroxy-6,8-dioxabicyclo [3.2.1] octan-4-one (OH-LGO) is avoided due to the catalytic specificity of alkene reductase (OYE 2.6) and present the best conversion rates.

In view of the high selectivity and activity of the enzyme in the catalytic process, meanwhile meet with the demand of green and sustainable catalytic process, more research should be accounted of in the upcoming development of cellulosic materials conversion.

## Conclusions and Perspectives

The transformation of cellulose to oxygenated and hydrocarbon chemicals was reviewed *via* several reaction routes over diverse multifunctional catalysts in aqueous phase. Sugars, alcohols, furfural, furan, and alkane chemicals can be obtained in one or two-step from cellulose conversion, which provide an alternative way to replace traditional fossil fuels with sustainable resources and potential solution for current environment problems. During the conversion process, complex reactions including hydrolysis, dehydration, isomerization, hydrogenolysis, and hydrogenation have high requirements for reaction systems and catalysts. (1) As to reaction solvent, the preferred reaction solvent for efficient conversion of cellulose to the downstream products for practical application is the aqueous phase, which is more available and economical than organic solvents or ionic liquids or other solvents. (2) For reaction conditions, high temperature and added extra gas (H_2_, O_2_) increased the cost of conversion process and exiting security issues, which would bring a lot of uncertainty for the reaction. (3) When it turns to catalysts, multifunctional catalysts were designed due to the complexity of the various reactions involved in the conversion process, in which different components of the catalysts have unique effects for specific reaction processes, for example, metal components are usual responsible for the hydrogenation, or hydrogenolysis of C-O or C-C bonds, or active sites of the reaction. (4) The reaction routes have always been valued in the study of cellulose aqueous-phase transformation, different reaction routes generally correspond to various types of reaction systems over diverse multifunctional catalysts, and the ultimate goal of the study is to achieve a clear understanding of the reaction and reduce the reaction steps as possible for energy-saving purposes. (5) Meet the requirements of green and sustainable catalysis, enzyme catalytic process of cellulosic materials has shown a giant potential for its high selectivity and activity, hence, looking for specific enzymes for specific reactions are of great significance. Overall, the research findings mentioned in this review provide a scientific basis for related subject study, which has broad prospects for future development in biomass conversion.

Although tremendous progresses have been achieved in the conversion of cellulose, there are still a lot of shortcomings and challenges that need to be solved in further study. (1) It can be confirmed that cellulose could be efficiently converted in aqueous phase, but many mineral acids (sulfuric acid, phosphoric acid, hydrochloric acid, etc.) or organic acids (formic acid, acetic acid, fatty acid, etc.) are involved in many aqueous phases, all of these acids has corrosive effects on reaction instruments and may cause water pollution problems, so pure water phase reaction should be the ultimate goal to pursue. (2) The efficient conversion of cellulose under milder conditions should be the focus of researchers. And taking the high oxygen content of cellulose and the presence of water in reaction system into consideration, can oxygen and hydrogen be supplied *in situ* during the reaction process? Those ways can both reduce the energy consumption and expenditure by a large margin. (3) The decisive steps in all kinds of reaction process should be defined in the beginning so that the catalyst can be designed more accurately, and researches on non-noble metals should be reinforced to minimize the use of precious metals in economic aspect. Furthermore, the interaction between different components of the multifunctional catalysts should be clearly understood in order to reduce the occurrence of side reactions and improve the yield of the target products. In particular, the hydrothermal stability and excellent catalytic activity of multifunctional catalysts are the most important things that should be the first to be considered to enable the full utilization of cellulose. (4) The reaction routes are still the key research object of cellulose aqueous phase transformation, the design of catalysts and the comprehension of reaction mechanisms would be a benefit from the premise of a clear reaction route. (5) The high cost, sensitivity to reaction temperature, and pH have become non-negligible factors restricting the application of enzymes in catalytic conversion of biomass. Therefore, further reducing the cost and improving the stability of the enzyme to adapt to various reaction conditions will be focused on in the next research.

## Author Contributions

HX and QL taking the lead in coordinating the review study and drafting the manuscript. CZ is responsible for obtaining the Scheme permission in the relevant cited references. XH, HW, CZ, SL, and ZX provided professional advice. CC, XZ, and QL participated in the work of manuscript revision. XZ, QL, and LM provided funding support. All authors read and approved the final manuscript.

## Conflict of Interest

The authors declare that the research was conducted in the absence of any commercial or financial relationships that could be construed as a potential conflict of interest.
